# Morpho-Functional Effects of Nonylphenol–Steroid Hormone Co-Exposure on Human Prostate PNT1A Cells

**DOI:** 10.3390/toxics14090755

**Published:** 2026-08-26

**Authors:** Aldo Mileo, Teresa Chianese, Stefania Boccia, Francesca Carrella, Benedetta Sgangarella Valvano, Rosaria Sciarrillo, Luigi Rosati, Lucia Capasso, Antonio De Luca, Anna Capaldo, Maria De Falco

**Affiliations:** 1 Department of Life Science, Health and Health Professions, Link Campus University, Via del Casale di San Pio V 44, 00165 Rome, Italy; a.mileo@unilink.it; 2 Department of Biology, University of Naples Federico II, Via Cinthia 26, 80126 Naples, Italy; teresa.chianese2@unina.it (T.C.); luigi.rosati@unina.it (L.R.); anna.capaldo@unina.it (A.C.); 3 Department of Mental and Physical Health and Preventive Medicine, Section of Human Anatomy, University of Campania “Luigi Vanvitelli”, Largo Madonna delle Grazie 1, 80138 Naples, Italy; stefania.boccia@unicampania.it (S.B.); antonio.deluca@unicampania.it (A.D.L.); 4 Department of Precision Medicine, University of Campania “Luigi Vanvitelli”, Vico L. De Crecchio 7, 80138 Naples, Italy; lucia.capasso2@unicampania.it; 5 Department of Science and Technology, University of Sannio, Via F. de Sanctis snc, 82100 Benevento, Italy; fcarrella@unisannio.it (F.C.); sciarrillo@unisannio.it (R.S.); 6 Department of Medicine and Health Sciences “Vincenzo Tiberio”, University of Molise, 86100 Campobasso, Italy; benedetta.sgangarella@unimol.it; 7 National Institute of Biostructures and Biosystems (INBB), 00136 Rome, Italy

**Keywords:** environmental pollution, nonylphenol, endocrine-disrupting chemicals, steroid receptors, prostate gland

## Abstract

Nonylphenol (NP) is a chemical compound belonging to the class of alkylphenols (APs), known for its widespread environmental distribution and endocrine-disrupting properties. It is commonly used in the production of detergents, pesticides, and plastic materials and, owing to these properties, it can accumulate in both aquatic and terrestrial ecosystems. As a xenoestrogenic compound, NP can bind steroid receptors, including estrogen receptors (ERs), thereby activating ER-dependent pathways. In this work, we investigated the effects of NP alone and in combination with the endogenous hormones 17β-oestradiol (E2) and/or testosterone (T) on a human non-tumoral prostate cell line (PNT1A). Cell viability and migration assays, together with analysis of ER expression and localization, were carried out to assess the xenoestrogenic activity of NP, particularly in the presence of E2 and T. Our results showed that NP retained its endocrine-disrupting features in the mixtures, positively affecting cell viability, except for the NP+E2 mixture, in which cell viability did not significantly differ from control, suggesting an antagonistic interaction between NP and E2. The mixtures also interfered with steroid receptor dynamics, affecting receptor expression and delaying receptor localization and activation kinetics. Moreover, all mixtures negatively affected cell migration compared with treatment with endogenous hormones alone. In conclusion, our results demonstrate that NP retains its xenoestrogenic behavior in mixture, inducing a significant alteration in prostate cell homeostasis.

## 1. Introduction

Nonylphenol (NP) is an industrial compound widely used as a non-ionic surfactant in plastic manufacturing, and in agricultural, personal care and household products. It is widely distributed in the environment, where it occurs at concentrations from 10^−6^ M to 10^−12^ M, owing to its high chemical stability and its tendency to accumulate in aquatic and terrestrial matrices such as rivers, seas, soils, groundwaters and sediments [1,2,3,4,5]. In line with Annex XVII of the REACH regulation, NP has been listed as a substance of very high concern (SVHC) owing to its reproductive toxicity and endocrine-disrupting action. Low-dose NP exposure can lead to adverse effects such as reproductive disorders, developmental abnormalities and increased susceptibility to hormone-related cancers [6,7]. NP has been shown to disrupt hormonal signaling pathways in several organisms, including humans. It mimics estrogens and modulates the intracellular trafficking of estrogen and androgen receptors (ERα, ERβ- and/or AR), promoting their translocation between the cytoplasm and nucleus and vice versa [8]. Exposure to NP has been linked with multiple adverse health effects, including dysregulation of the immune and neuroendocrine systems [7,9]. NP can impair cell cycle regulation in several cell types, including neuronal stem cells [10], human gastric adenocarcinoma cells (AGS) [11], and bronchial epithelial cell lines [12]. Although NP can deregulate the physiology of several organs, the reproductive system is particularly sensitive to NP exposure. It has been demonstrated that NP caused a marked, dose-dependent reduction in cell viability and increase in apoptosis in murine GC-1 spermatogonia cells [13] and exhibited a dose- and time-dependent anti-proliferative effect in rat Sertoli cells (TM4) [14].

A key target of endocrine-disrupting chemicals (EDCs) is the prostate gland, which, like the breast gland, is under the control of both female and male steroid hormones. For this reason, the prostate gland is highly responsive to xenoestrogenic compounds such as NP [15]. Specifically, prostate physiology is influenced by the estrogen/androgen ratio (E2/T), which can be considered a key indicator of prostate health status. An increase in E2 levels, resulting from reduced T concentrations and/or the presence of xenoestrogens, promotes prostate cell proliferation. This condition may contribute to the development of benign prostatic hyperplasia and, in more severe cases, prostate cancer. Prostate cancer is a multifactorial disease associated with several established risk factors including aging, lifestyle habits, family history, obesity, hypertension, genetic predisposition, antiretroviral therapy and unknown etiological factors [16,17]. In recent decades, environmental pollution has also emerged as a major contributor to cancer risk. Indeed, the increasing incidence of prostate cancer in industrialized countries suggests a possible association between environmental pollution and prostate carcinogenesis [18]. Prostate cancer is the second leading cause of cancer-related deaths among men worldwide [19] and in the U.S.A. [20]. It is also the most frequently diagnosed male cancer in the U.S.A., accounting for approximately 26% of all newly diagnosed cancers and 11% of cancer-related deaths, second only to lung cancer (22%) [21].

NP can disrupt normal prostate endocrine function, leading to prostatic hyperplasia and potentially contributing to prostate cancer development [15]. The mechanism through which NP exerts its effects involves interference with androgen and estrogen receptor signaling pathways, which are crucial for maintaining prostate health and function [22]. We have previously shown that NP exerts cancer-promoting effects by altering cell cycle progression through ERα translocation from the cytoplasm to the nucleus. ERα nuclear localization enhances the gene expression of cyclin D1, cyclin E and Ki67, and upregulates key genes involved in the inflammatory process, in a non-tumorigenic prostate cell line (PNT1A) [23]. After NP treatment, IL-8 and IL-1β mRNA levels increased by more than 50%. These findings indicate that NP triggers stronger inflammatory responses than E2 in PNT1A cells [23]. We have also shown that NP exerts effects on the androgen-dependent prostate tumor cell line (LNCaP), inducing cell proliferation, S-phase progression, and increased ERα expression with translocation from the cytoplasm to the nucleus [24].

Building on our previous work demonstrating that NP could promote proliferation by activating the ERα-dependent pathway in PNT1A cells [23,24], the aim of the present study was to investigate the effects of mixture of NP with E2 and/or T and their related mechanisms, which remain poorly understood. Specifically, the present study addresses a critical gap in EDC research by investigating the “cocktail effects” arising from the interaction between NP and endogenous steroid hormones. Because real-life exposure rarely involves a single contaminant, combined exposures represent one of the greatest challenges for EDC risk assessment. We therefore evaluated the impact of these interactions on cell viability, steroid receptor dynamics, and migratory capacity in PNT1A cells.

## 2. Materials and Methods

### 2.1. Experimental Setup

Testosterone (T, 86500-5G), 17β-oestradiol (E2, E8875-1G) and 4-nonylphenol (NP, 74430-1L) were purchased from Sigma-Aldrich (St. Louis, MO, USA) and dissolved in dimethyl sulfoxide (DMSO).

In agreement with previous studies [25], preliminary toxicity assays were carried out to establish the dose–response relationship. Following established practice for EDC mixture studies [26], each compound was tested at concentrations ranging from 10^−6^ M to 10^−12^ M after 24 h of exposure. For each compound, the concentration producing the greatest statistically significant change in cell viability compared with the control was selected for the mixture experiments (NP and E2 at 10^−6^ M; T at 10^−8^ M). Because none of the compounds showed a clear dose–response relationship, an effect-based criterion was preferred over the use of the highest tested concentration. Table 1 reports the experimental classes used in this work [24,25].

### 2.2. Cell Cultures

Immortalized non-tumoral prostate cells (PNT1A, ECACC 95012614) were cultured in phenol red-free RPMI 1640 medium supplemented with 10% charcoal-stripped FBS (fetal bovine serum), 1% L-glutamine, and 1% penicillin/streptomycin, and maintained in an incubator at 37 °C in a 5% CO_2_. Phenol red-free medium and charcoal-stripped serum were used to minimize interference from the estrogenic activity of phenol red and from steroid hormones present in the serum. At 70–80% confluence, cells were harvested and reseeded into new flasks. At the time of treatment, the serum concentration was lowered to 1%. To exclude solvent-related effects, the DMSO (vehicle) concentration was kept constant at 0.1% (*v*/*v*) across all experimental conditions, including single compounds, mixtures and control. Consequently, control cells were exposed to 0.1% DMSO, matching the vehicle concentration present in each treatment. Cells were observed daily under an inverted microscope.

### 2.3. MTT Assay

The effects of NP, alone and in mixture with endogenous hormones, on PNT1A cell proliferation were evaluated using the 3-(4,5-dimethylthiazol-2-yl)-2,5-diphenyltetrazolium bromide (MTT) assay (Sigma-Aldrich, St. Louis, MO, USA) [27]. Briefly, cells were seeded at a density of 5 × 10^3^ cells/well in 96-well plates. After starvation (1% FBS) and 24 h of treatment with the mixtures shown in Table 1, 10 µL of MTT salt solution (5 mg/mL) was added to the culture medium, reaching a final concentration of 0.5 mg/mL. After 2 h of incubation at 37° C in 5% CO_2_, the formazan crystals were dissolved in DMSO. The absorbance of each sample at 570 nm was measured using a Synergy H1 microplate reader (Biotek Instruments, Winooski, VT, USA). Cell viability was expressed as follows: (treated Abs_570_ − blank)/(control Abs_570_ − blank) × 100.

### 2.4. Western Blotting

After treatment (Table 1) for three different time points (30 min, 2 h and 4 h), total cell proteins were extracted using RIPA buffer (50 mM Tris, 150 mM NaCl, 0.1% SDS, 0.5% sodium deoxycholate, 1 mM NaF, 1% NP-40, 10 mM EDTA) enriched with protease inhibitors (Sigma-Aldrich, St. Louis, MO, USA). After protein quantification by BCA protein assay, 4 X Laemmli buffer (50 mM Tris-HCl pH 6.8, 2 g/100 mL SDS, 10% *v*/*v* glycerol, 0.1 g/100 mL bromophenol blue) was added to 40 µg of protein [28]. Proteins were boiled at 95° C for 5 min, separated by 10% SDS-PAGE and transferred onto a PVDF membrane. Membranes were then cut into horizontal strips according to the molecular weight of each target protein before antibody incubation. This approach allowed the simultaneous detection of ERα, ERβ, AR and GAPDH from the same protein extracts. The strips were incubated with blocking buffer (TBS, 0.1% Tween-20, 5% non-fat milk) for 1 h at room temperature and then incubated overnight at 4 °C with primary antibodies diluted in TBS-Tween-20 with 3% non-fat milk. The following day, the membrane was incubated with secondary antibodies. The primary and secondary antibodies used, and their dilutions, are listed in Table 2. Protein molecular weights were estimated using the Opti-Protein Ultra Marker (cat. no. G623; Applied Biological Materials Inc., Richmond, BC, Canada), a pre-stained protein ladder covering a range from 6.5 to 270 kDa. After incubation, protein bands were detected using a chemiluminescent HRP substrate (Cat. No. E-IR-R307, Elabscience Bionovation Inc., Houston, TX, USA) and visualized with a chemiluminescence scanner (ChemiDoc Imaging System, Bio-Rad Laboratories, Hercules, CA, USA). Band intensities were quantified using ImageJ 1.54f software (NIH, Bethesda, MD, USA). Optical density (OD) values of the bands were normalized to GAPDH levels to correct for differences in protein loading.

### 2.5. Immunofluorescence Assay

Immunofluorescence assays were performed to determine the localization of ER and AR receptors in PNT1A cells exposed to NP and endogenous hormones. Briefly, PNT1A cells were seeded on round coverslips placed in 6-well plates at a density of 2.0 × 10^5^ cells/well. Treatments (Table 1) were applied at three time points (30 min, 2 h and 4 h) to identify receptor localization. Cells were fixed in 4% PFA for 15 min at room temperature and permeabilized with 0.25% PBS-Triton (PBS-T). Non-specific binding sites were blocked by incubation with 2% BSA in PBS-T for 1 h [29]. Cells were then incubated with primary antibody overnight at 4 °C and subsequently with secondary antibody for 1 h at room temperature. The primary and secondary antibodies and dilutions used are listed in Table 3. Nuclei were stained with DAPI (4′,6-diamidino-2-phenylindole) at a dilution of 1:1000. Coverslips were finally mounted on slides with a 1:1 glycerol/PBS 1× solution. The negative control was obtained by omitting the primary antibodies. All observations were performed with an FluoView FV3000 confocal laser scanning microscope (Olympus, Tokio, Japan). Immunofluorescence analyses were performed in triplicate, with different fields chosen for data analysis. The nuclear cytoplasmic fluorescence ratio of ERα, ERβ and AR (N/C) was carried out by ImageJ and quantified using Fiji software (NIH, Bethesda, MD, USA; version 1.54). Nuclei were segmented using the DAPI channel by automatic thresholding (Otsu method), followed by watershed separation of adjacent nuclei. The cytoplasm was defined by a perinuclear band around each nucleus. For each cell, the mean receptor intensity was measured in both compartments, and their ratio was calculated (ratio > 1 = predominant nuclear localization).

### 2.6. Wound Healing Assay

PNT1A cells were seeded in 24-well plates. Once 100% confluence was reached, a “wound” was created in the middle of the cell monolayer using a sterile P1000 tip. After PBS washing, cells were exposed to the experimental mixtures for 24 h. Cell migration was monitored using a JuLI™ Stage live-cell imaging system (NanoEnTek, Seoul, Republic of Korea), an inverted microscope that allowed cell migration to be followed by time-lapse imaging. Image analysis was carried out using ImageJ with the “Wound Healing Size Tool”. The average migration speed after 24 h was obtained as the ratio of distance to time.

### 2.7. Statistical Analysis

Data are presented as mean values ± SEM. Statistical analysis was conducted using GraphPad Prism software version 8.0.2 (GraphPad Software, San Diego, CA, USA) The significance of differences was assessed via ANOVA followed by Dunnett’s multiple comparison test. To formally evaluate interaction effects in the mixtures, viability data were additionally analyzed by two-way ANOVA, with each compound as a fixed factor; a significant interaction term was interpreted as evidence of a non-additive (antagonistic or synergistic) interaction. Results were considered statistically significant at *p* < 0.05. All experiments were performed in triplicate, with three independent biological replicates and technical replicates for each condition.

## 3. Results

### 3.1. Effects of NP, E2, T and Their Mixtures on PNT1A Cell Viability

**Figure 1:**
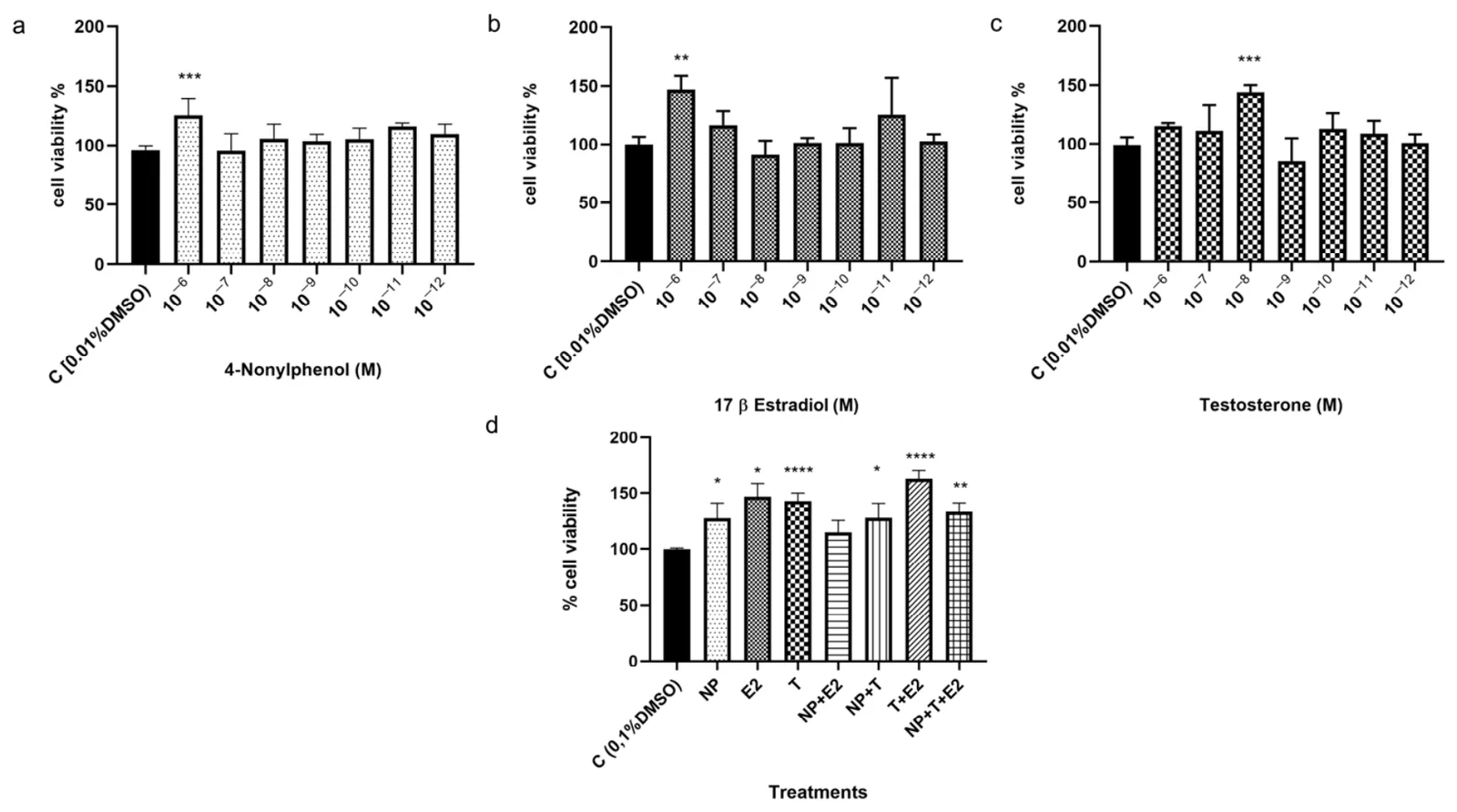
MTT assay after 24 h of treatment with 4-nonylphenol (NP) (**a**), 17β-oestradiol (E2) (**b**), and testosterone (T) (**c**). Cell viability was significantly increased by NP (10^−6^ M), E2 (10^−6^ M) and T (10^−8^ M), respectively. When used in combination, the mixtures NP+T and NP+T+E2 significantly increased cell viability, whereas NP+E2 did not differ significantly from the control (**d**). Asterisks indicate significant differences versus control (Dunnett’s test: * *p* < 0.05, ** *p* < 0.01 *** *p* < 0.001 **** *p* < 0.0001). Interaction effects in the mixtures were evaluated by two-way ANOVA (*n* = 3), which revealed significant antagonistic NP × E2 (F (1,8) = 25.2, *p* = 0.001) and NP × T (F (1,8) = 12.5, *p* = 0.008) interactions.

To evaluate the significant effect on cytotoxicity, an MTT assay was carried out after 24 h of treatment. PNT1A cells were preliminarily exposed to NP, E2, or T at concentrations ranging from 10^−6^ M to 10^−12^ M. Physiological, environmental, and supra-physiological concentrations were evaluated to establish a dose–response relationship. None of the tested compounds showed a clear dose-dependent relationship. Moreover, no cytotoxic effects were observed, even at the highest concentration (10^−6^ M). NP and E2 significantly increased cell viability, especially at 10^−6^ M, in agreement with previous findings [23]. T induced a similar modulation, although at a lower concentration (10^−8^ M) (Figure 1a–c). Mixtures were subsequently formulated using the concentrations that produced the most significant effect for each compound, to investigate potential cocktail effects. After 24 h of treatment, NP+T and NP+T+E2 significantly increased cell viability. In contrast, NP+E2 did not show any significant effect compared with the control, although this mixture slightly increased cell viability. Moreover, two-way factorial ANOVA confirmed that the interactions between NP and the endogenous hormones were antagonistic: in both the NP+E2 and NP+T mixtures, cell viability was significantly lower than that predicted by an additive combination of the single compounds (Figure 1d).

### 3.2. Time-Dependent Modulation of Estrogen and Androgen Receptor Expression by NP, E2, T and Their Mixtures

**Figure 2:**
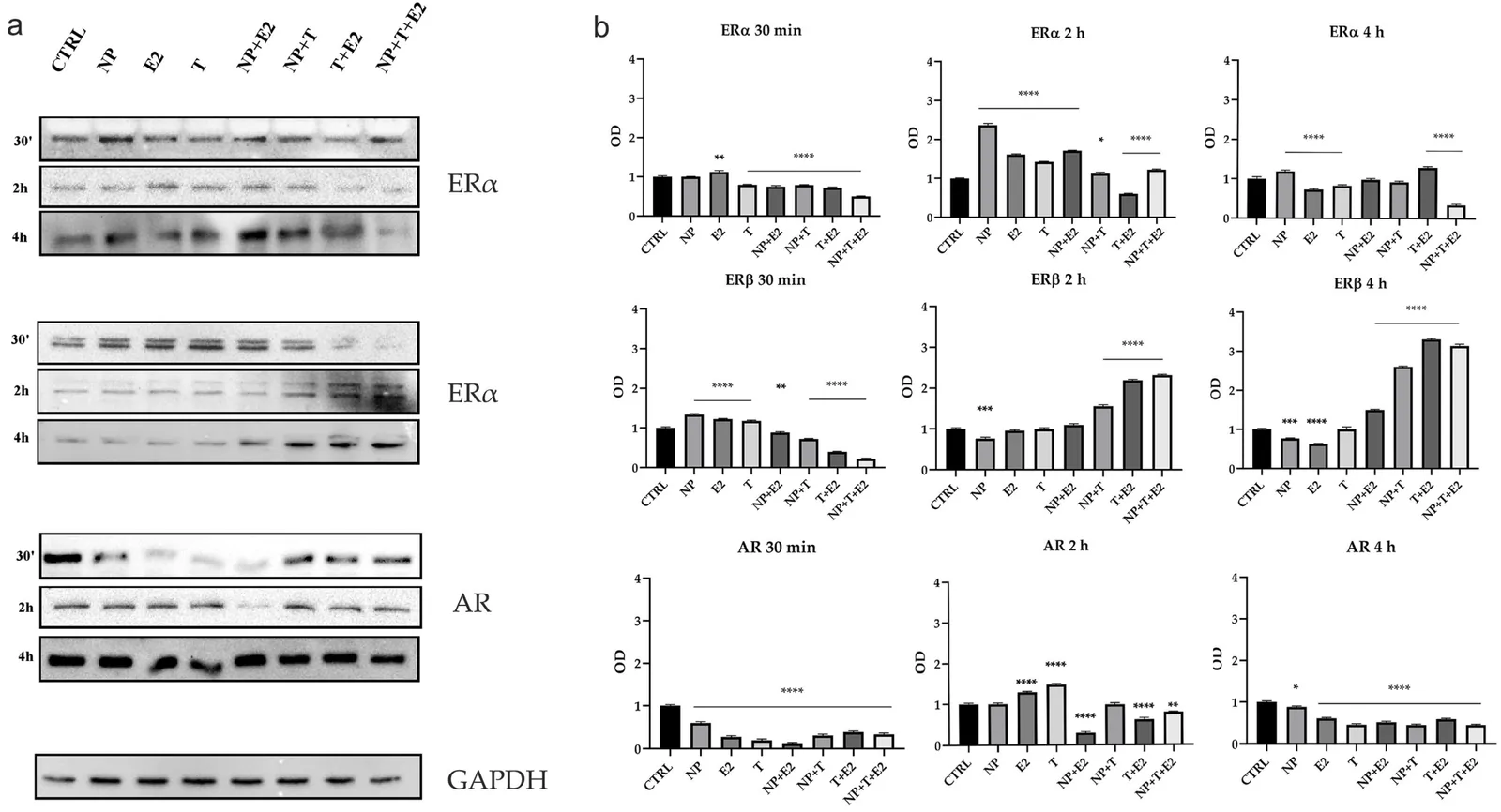
Western blot analysis of ERα, ERβ and AR after 30 min, 2 h and 4 h of NP, E2 and T treatment, alone and in mixture. Membranes were cut into horizontal strips according to the molecular weight of each target prior to antibody incubation; (AR ≈ 110 kDa, ERα ≈ 66 kDa, ERβ ≈ 56 kDa, GAPDH ≈ 37 kDa) (**a**). The graphs represent the optical density (OD) ratio of ERs and AR normalized to the OD of GAPDH (**b**). (Dunnett’s test: * *p* < 0.05 ** *p* < 0.01, *** *p*< 0.001 **** *p* < 0.0001).

To compare the estrogen and androgen expression levels with their localization, the assays were carried out after 30 min, 2 and 4 h of treatment. The protein expression of the two estrogen receptor isoforms (ERα, ERβ) and of the androgen receptor (AR) was assessed after 30 min, 2 h and 4 h of treatment, to verify whether the treatments could transiently affect the protein levels of these receptors (Figure 2a,b). After 30 min, only E2 slightly increased ERα expression, whereas NP showed no significant effect compared with the control. In contrast, all mixtures reduced ERα expression. After 2 h, NP, and NP+E2 markedly increased ERα levels, whereas NP+T induced only a modest upregulation. Among all treatments, NP produced the highest ERα expression. After 4 h of treatment, NP induced a slight upregulation of ERα compared with the control; NP+E2 and NP+T did not show any difference from the control, while NP+T+E2 unexpectedly inhibited ERα expression. NP slightly increased ERβ expression after 30 min, whereas E2 and T induced weaker effects. In contrast, all the mixtures transiently downregulated ERβ at 30 min. After 2 and 4 h, the mixtures increased ERβ expression, whereas NP alone reduced receptor levels. After 30 min, all treatments reduced AR expression compared with the control, although NP showed the highest OD value. After 2 h, NP showed no significant effect compared with the control, whereas both E2 and T significantly increased AR expression. Conversely, all mixtures downregulated AR expression, particularly NP+E2. After 4 h, the trend resembled that observed at 30 min, with a general downregulation of AR compared with the control (Figure 2a,b).

### 3.3. NP, E2, T and Mixtures Differentially Modulate the Subcellular Localization of ERα, ERβ and AR

To investigate if NP, alone and in mixture, could promote estrogen and/or androgen receptor translocation, immunofluorescence assays were carried out after 30 min, 2 and 4 h of treatment. These time points were selected based on the kinetics of steroid receptor nuclear translocation: ERα and AR typically translocate to the nucleus within 15–30 min of high-affinity ligand exposure, reach a peak of nuclear accumulation at 1–2 h, and may redistribute or undergo proteasomal degradation by 4 h [27]. The 4 h endpoint was specifically included to capture the potentially delayed receptor dynamics expected from NP, given its lower receptor affinity compared with endogenous hormones [23].

#### 3.3.1. ERα Localization

**Figure 3:**
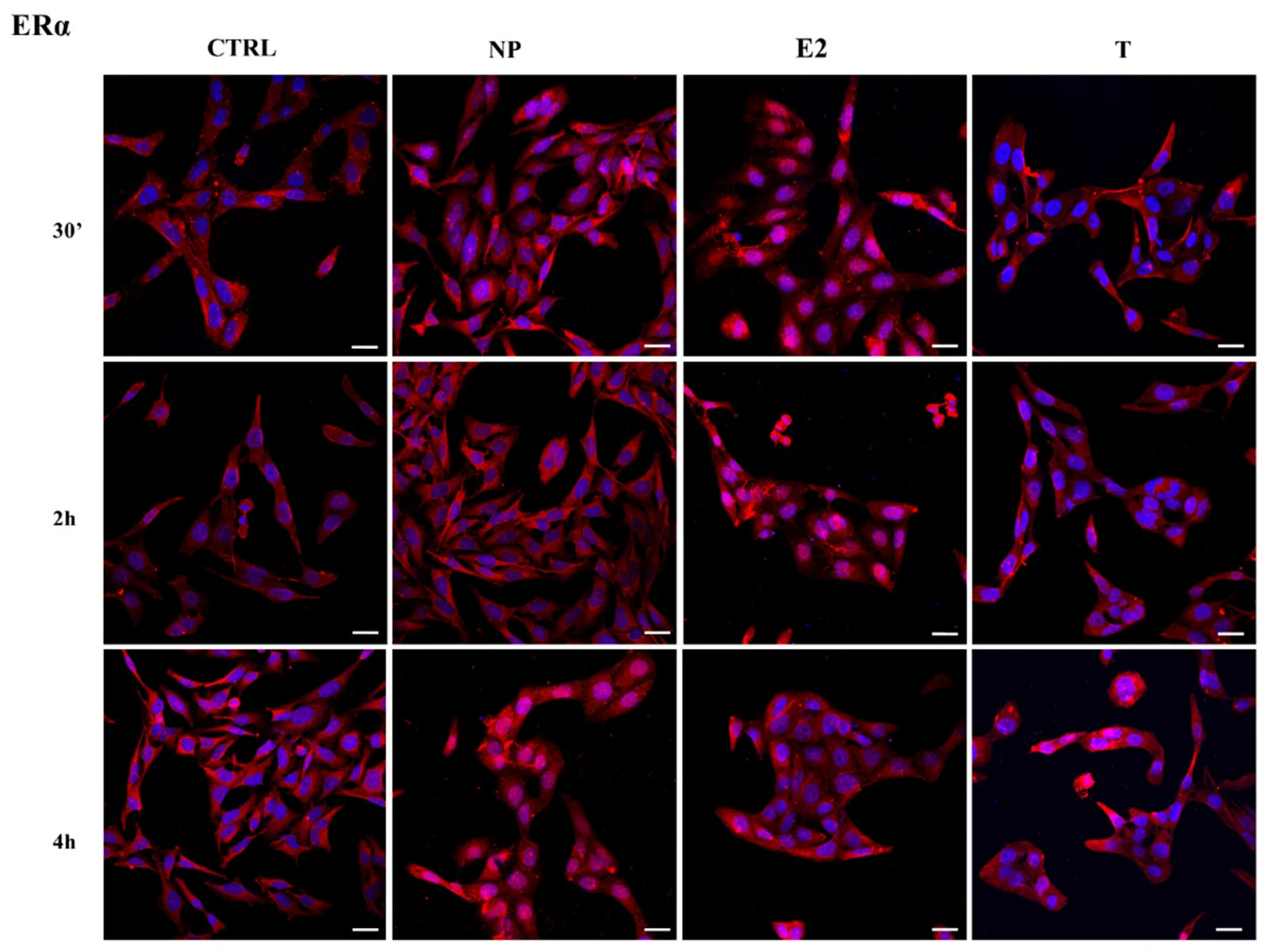
Localization of ERα after 30 min, 2 h and 4 h of exposure to DMSO, NP, E2 and T. ERα was localized in the nucleus after 30 min of E2 treatment, while NP induced late ERα nuclear translocation after 4 h of treatment compared with DMSO exposure; T did not induce any ERα translocation. ERα immunoreactivity is shown in red (Alexa Fluor 594), while nuclei were counterstained in blue with DAPI; samples were analyzed by immunofluorescence. Scale bar: 10 µm.

**Figure 4:**
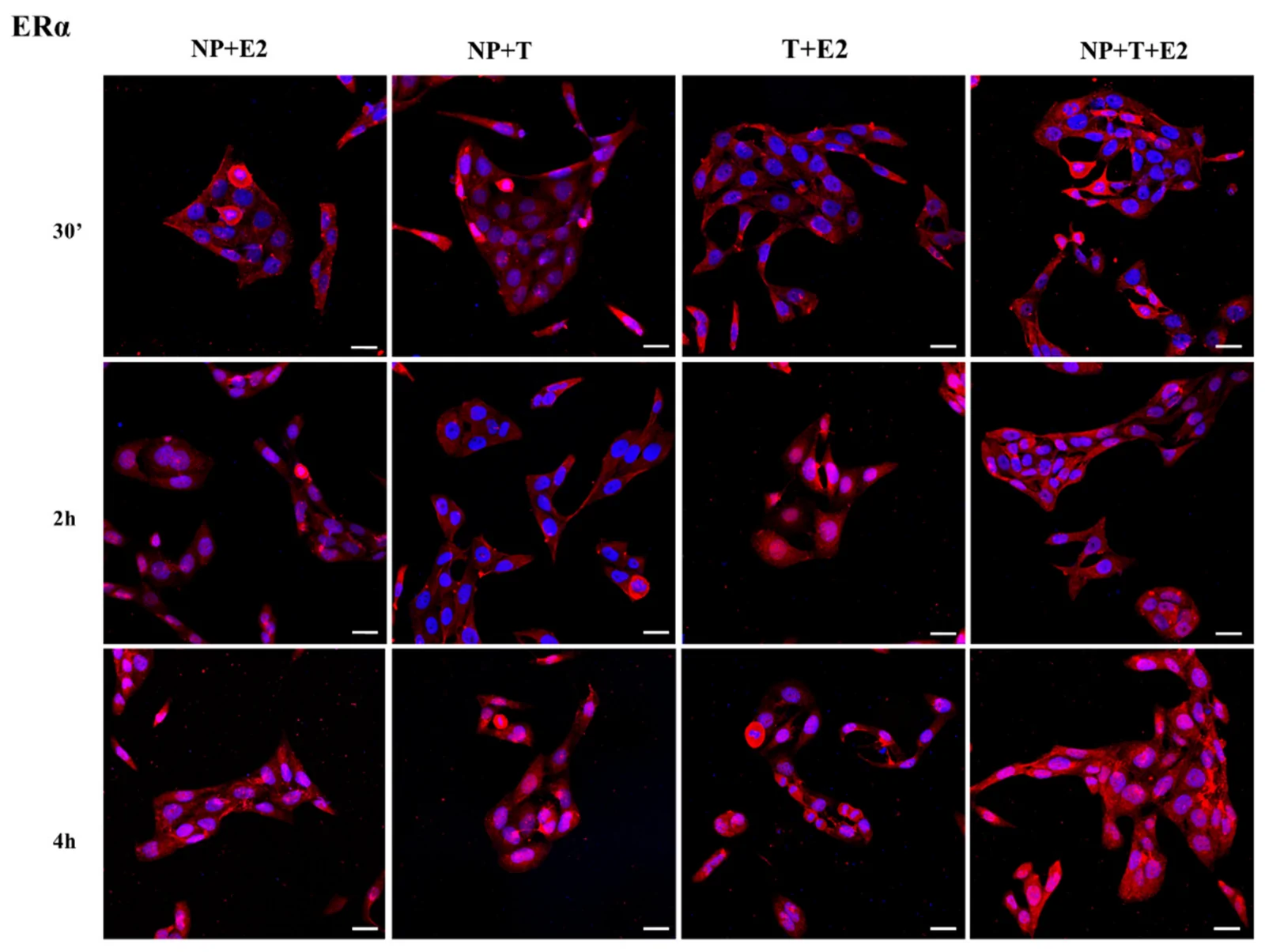
Localization of ERα after 30 min, 2 h and 4 h of exposure to NP+E2, NP+T, T+E2 and NP+T+E2. ERα nuclear translocation was observed after 2 and 4 h of NP+E2 and T+E2 treatment, and after 4 h of NP+T and NP+T+E2 treatment. ERα immunoreactivity is shown in red (Alexa Fluor 594), while nuclei were counterstained in blue with DAPI; samples were analyzed by immunofluorescence. Scale bar: 10 µm.

Immunofluorescence analysis of ERα was carried out to determine whether NP, alone or in mixture, was able to activate ERα-dependent pathways: all treatments induced ERα translocation. In the control, ERα was consistently localized in the cytoplasm (Figure 3). E2 induced translocation after 30 min and 2 h of treatment, owing to its greater affinity for ERα (Figure 3). In contrast, NP alone induced ERα translocation only after 4 h of treatment (Figure 3). T never induced ERα translocation to the nucleus (Figure 3). The NP+E2 mixture activated ERα translocation after 2 h of treatment, with the receptor remaining in the nucleus after 4 h (Figure 4). NP+T and NP+T+E2 followed the same trend as NP alone, inducing ERα translocation only after 4 h of treatment (Figure 4). The T+E2 mixture showed ERα translocation after both 2 and 4 h of treatment (Figure 4). Quantification of the N/C ratio confirmed this pattern: E2 induced a transient nuclear localization of ERα that declined by 4 h, whereas the NP+E2 mixture produced a nuclear retention up to 4 h (Supplementary Figure S1).

#### 3.3.2. ERβ Localization

**Figure 5:**
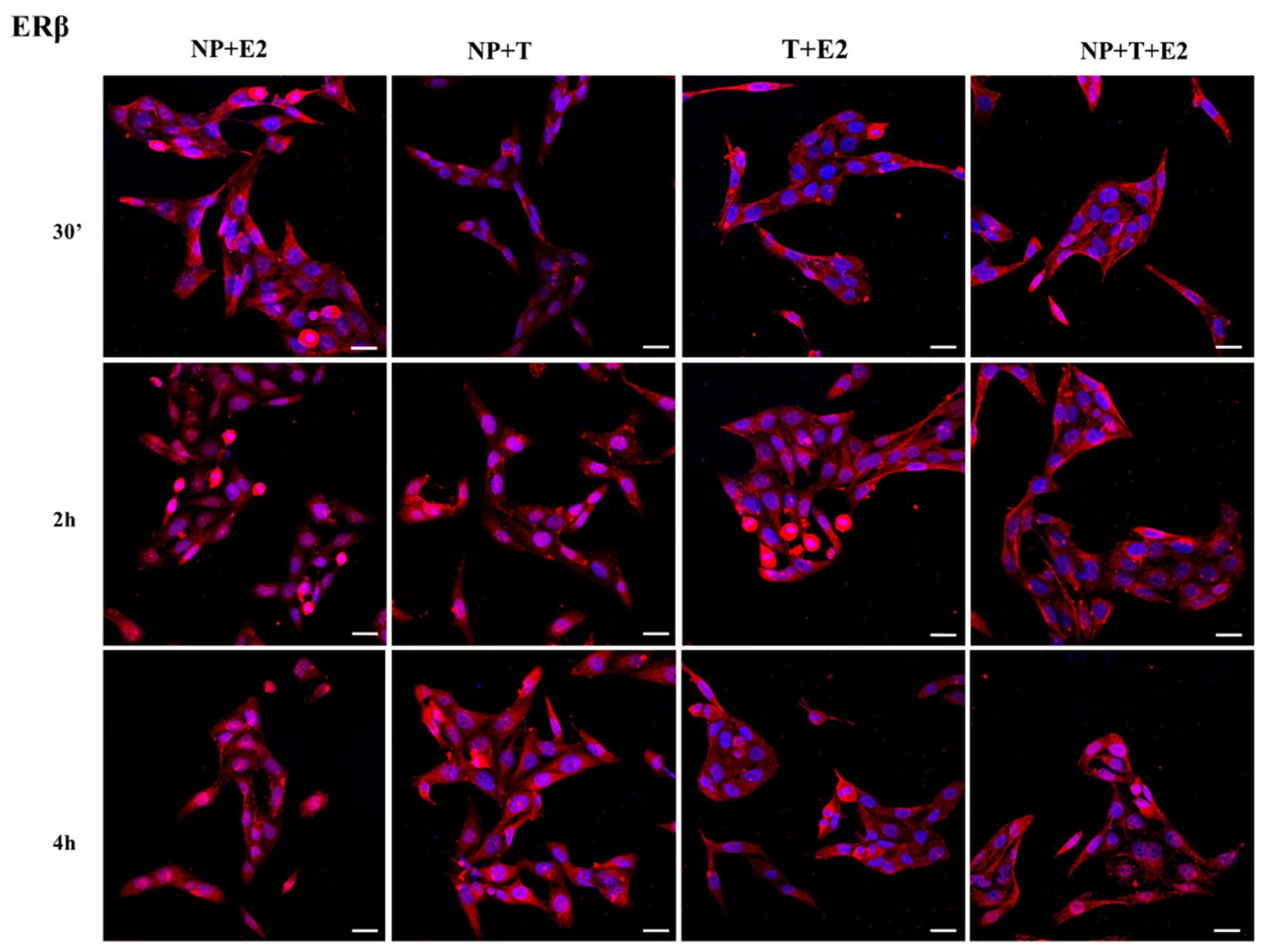
Localization of ERβ after 30 min, 2 h and 4 h of exposure to NP+E2, NP+T, T+E2 and NP+T+E2. ERβ was localized in the nucleus after 2 and 4 h of treatment. After NP+T exposure, ERβ showed a cyclic behavior: localized in the cytoplasm after 30 min, translocated to the nucleus after 2 h, and returned to the cytoplasm after 4 h of treatment. After T+E2 treatment, ERβ remained in the cytoplasm at all time points, whereas NP+T+E2 induced ERβ nuclear localization after 4 h of treatment. ERβ immunoreactivity is shown in red (Alexa Fluor 594), while nuclei were counterstained in blue with DAPI; samples were analyzed by immunofluorescence. Scale bar: 10 µm.

Immunofluorescence performed to localize ERβ in PNT1A cells exposed to different substances showed a dynamic different from that of the α isoform. ERβ was consistently localized in the cytoplasm both in controls and in treatments with a single compound (Supplementary Figure S1); although NP showed estrogenic activity, it was not able to translocate ERβ, showing no difference from the control (Supplementary Figure S1). When cells were treated with the mixtures, however, cytoplasmic/nuclear translocation occurred. NP+E2 induced nuclear localization after 2 and 4 h, indicating a weak affinity for the receptor (Figure 5); NP+T shifted ERβ to the nucleus after 2 h of treatment, with the receptor returning to the cytoplasm after 4 h (Figure 5). NP+T+E2 induced a delayed ERβ translocation only after 4 h of treatment (Figure 5). Quantification of the N/C ratio confirmed that ERβ remained largely cytoplasmic after the single treatments and translocated to the nucleus in the mixtures, especially after NP+E2 treatment (Supplementary Figure S2).

#### 3.3.3. AR Localization

**Figure 6:**
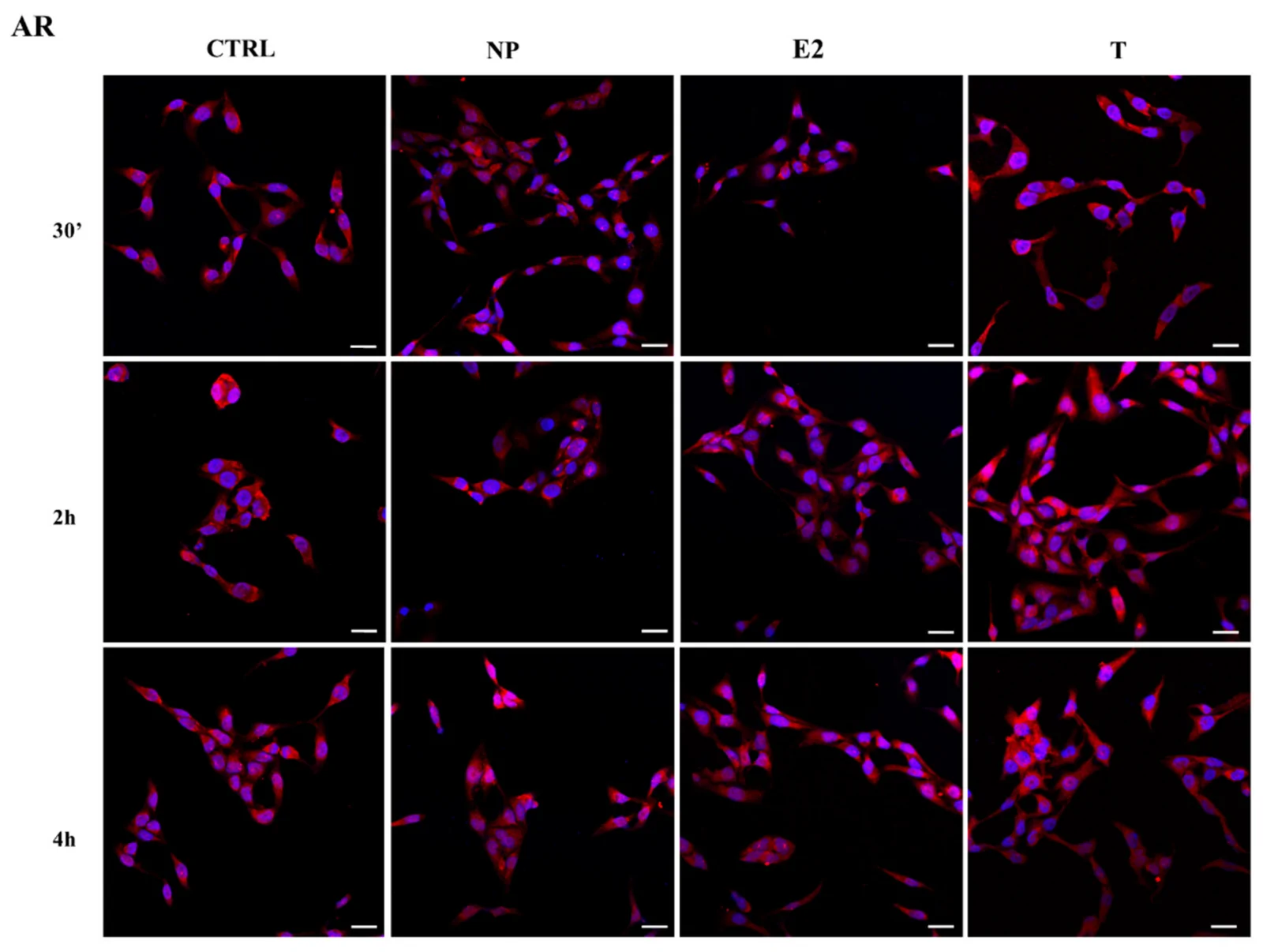
Localization of AR after 30 min, 2 h and 4 h of exposure to DMSO, NP, E2 and T. After NP exposure, AR showed a cyclic behavior, being localized in the nucleus after 30 min, translocating to the cytoplasm after 2 h, and returning to the nucleus after 4 h of treatment. After E2 treatment, AR was predominantly localized in the cytoplasm at all time points, with weak nuclear localization after 30 min and 4 h. After T treatment, AR was predominantly localized in the nucleus after 30 min and 2 h, returning to the cytoplasm after 4 h of treatment. AR immunoreactivity is shown in red (Alexa Fluor 594), while nuclei were counterstained in blue with DAPI; samples were analyzed by immunofluorescence. Scale bar: 10 µm.

**Figure 7:**
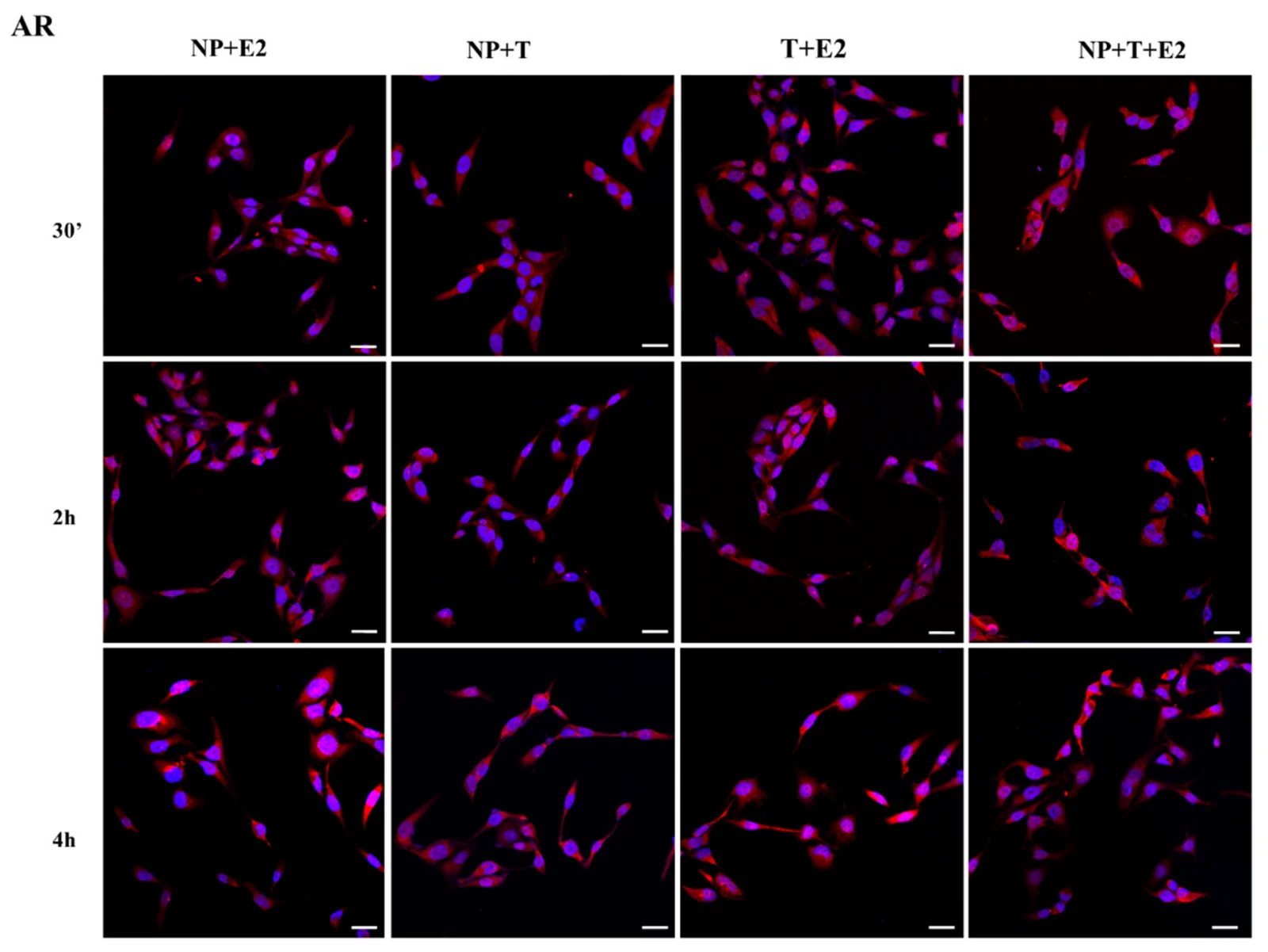
Localization of AR after 30 min, 2 h and 4 h of exposure to NP+E2, NP+T, T+E2 and NP+T+E2. After NP+E2 treatment, AR was localized in the nucleus after 30 min and 2 h, returning to the cytoplasm after 4 h; NP+T induced AR cytoplasmic localization at all time points; T+E2 exposure induced AR nuclear localization after 2 and 4 h; NP+T+E2 promoted AR localization in the nucleus after 30 min, a shift to the cytoplasm after 2 h, and co-localization in both nuclear and cytoplasmic compartments after 4 h. AR immunoreactivity is shown in red (Alexa Fluor 594), while nuclei were counterstained in blue with DAPI; samples were analyzed by immunofluorescence. Scale bar: 10 µm.

The AR receptor showed behavior different from that of the ERs. In the control, AR showed co-localization in the nucleus and cytoplasm, being localized in the nucleus after 4 h (Figure 6). NP induced AR nuclear localization after 30 min of treatment and again after 4 h (Figure 6). Although E2 is the hormone specifically able to activate ERα, it also showed the capability to bind the AR receptor, which was weakly localized in the nucleus after 30 min and 4 h of treatment (Figure 6). On the contrary, T induced nuclear translocation after 30 min and 2 h, suggesting a greater affinity for the receptor (Figure 6), with AR returning to the cytoplasm after 4 h of treatment. Regarding the mixtures, NP+E2 showed the same behavior as T treatment, translocating AR to the nucleus after 30 min and 2 h of treatment (Figure 7). NP+T did not show any translocation activity, with the receptor remaining in the cytoplasm throughout (Figure 7). T+E2 induced AR translocation after 2 and 4 h of treatment (Figure 7). NP+T+E2 showed the same behavior as NP alone, inducing nuclear AR translocation after 30 min and after 4 h (Figure 7). Quantification of the N/C ratio was consistent with the more diffuse, dual localization of AR, which showed comparatively high nuclear/cytoplasmic values and greater variability across conditions (Supplementary Figure S1).

### 3.4. NP Reduces Wound Closure and Antagonizes the Promigratory Effect of Endogenous Hormones

**Figure 8:**
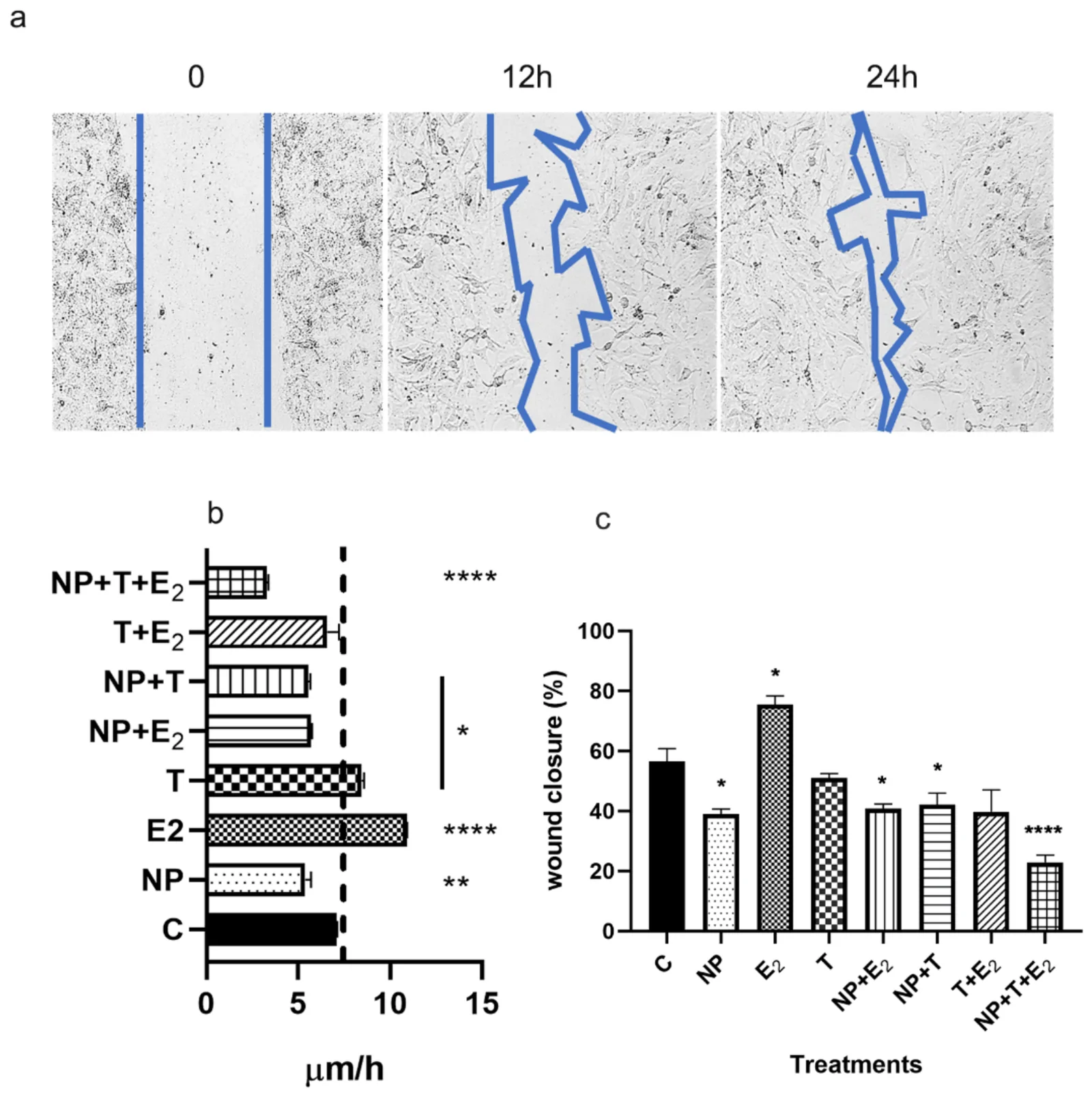
Wound healing assay after 24 h of treatment. (**a**) Representative phase-contrast micrographs of the scratched PNT1A monolayer for each experimental class, acquired at 0 h and 24 h; the dashed lines indicate the wound edges. (**b**) Quantification of the mean wound closure rate (µm/h) for each treatment. It was reduced in all mixtures compared with the control, while E2 and T alone increased it. The vertical dashed line represents the mean migration rate of the control (**c**) Wound closure after 24 h of treatment: NP, alone and in mixtures decreased the wound closure. Details in the text (Dunnett’s test: * *p* < 0.05; ** *p* < 0.01; **** *p* < 0.0001).

To evaluate the effects on cell migration, the wound healing assay was carried out after 24 h of treatment, measuring the reduction in the cell-free area. The analyses showed that all mixtures reduced the rate of wound closure of PNT1A cells compared with the control. The endogenous hormones E2 and T increased the migration rate compared with the control, whereas the presence of NP in the mixtures counteracted the migratory effect of both E2 and T. Consistently, the same trend was observed when migration was expressed as the percentage of wound closure after 24 h of treatment: the presence of NP reduced wound closure compared with the control, with the strongest effect in the ternary mixture NP+T+E2 (Figure 8a–c).

## 4. Discussion

EDCs are ubiquitous compounds of natural or anthropogenic origin. Owing to their structural similarity to endogenous hormones, they can interfere with endocrine signaling and induce adverse biological responses, especially during developmental stages, when tissues are most sensitive to hormonal perturbation. The alkylphenols, often used as surfactants or in the production of detergents, paints, pesticides, and personal care products, belong to this large group of substances [30]. NP is among the main representatives of EDCs, and its xenoestrogenic activity and interference with the endocrine system are well documented [23,24,31,32]. Like many EDCs, its effect can be defined as non-monotonic, since NP exerts its effect at a well-defined dose rather than following a canonical dose–response relationship. Owing to its chemical–physical structure, it can interact with hormone receptors, in particular the estrogen and androgen receptors (ERs, ARs), inducing different cellular responses.

In this work, using the non-tumoral prostate cell line PNT1A as a model, we aimed to deepen our knowledge about the effects of NP, focusing our attention on the mixture with the endogenous hormones E2 and/or T, whose function is fundamental to prostate physiology. In MTT assays, NP, E2, and T, alone or in mixture, increased cell viability, suggesting that these substances can positively affect cell metabolic activity, a response compatible with, although not demonstrative of, an increased number of viable cells. None of the three compounds showed a canonical dose–response relationship. Therefore, the concentrations selected for the mixture experiments were not the highest non-cytotoxic doses, but those that produced the greatest statistically significant biological response individually. A statistically significant increase in cell viability was recorded following treatment with NP+T and NP+T+E2. In contrast, no significant effect was observed after NP+E2 treatment, with cell viability not differing from the control; this effect suggested a potential interference between NP and E2. The increase in cell viability observed in PNT1A cells agrees with previous reports showing that NP exerts cell-type-specific effects. In gastric epithelial AGS cells, NP promotes proliferation [11]. In contrast, it induces apoptosis in SCM1 gastric cancer cells and in neural stem cells through caspase activation and disruption of cell cycle regulation [10,12]. This dual, tissue-specific outcome indicates that the biological effect of NP is strongly dependent on the receptor context of the target cell. In hormone-sensitive tissues, and particularly in the prostate, NP appears to act predominantly as a xenoestrogen driving proliferation: in prostate adenocarcinoma LNCaP cells, NP induces proliferation and S-phase progression via estrogen-related pathways [24], and we have previously reported comparable estrogen-like, proliferative effects in the non-tumorigenic PNT1A model [23]. Since the MTT assay quantifies mitochondrial reductase activity, the observed increase could reflect a larger number of viable cells, a higher metabolic rate per cell, or both; direct proliferation assays, such as cell cycle analysis, Ki-67 immunodetection or crystal violet staining, will therefore be required to confirm whether the NP+T and NP+T+E2 mixtures increase cell number. The present results extend this pattern to NP–hormone mixtures, confirming that the prostate epithelium responds to NP mainly through an estrogenic, growth-promoting response rather than through cytotoxicity. To investigate the role of steroid receptors in cell metabolism effects, we performed Western blot and immunofluorescence to detect protein expression levels and cellular localization of ERs and AR. NP, E2, and T, alone or in binary or tertiary combinations, had a transient influence on the expression levels of ERs and AR. Steroid hormone receptors are dynamically regulated proteins. ERα undergoes continuous proteasome-mediated turnover [33], and its levels are altered by xenoestrogens [34], whereas AR constantly shuttles between cytoplasm and nucleus [35]. Their total levels therefore fluctuated slightly even in control conditions, as also seen in our previous work on this model [23]. NP induced a transient increase in ERα expression, especially after 2 h of treatment, showing an estrogen-like trend but with a more pronounced effect than E2. In the mixtures, NP induced an increase in ERα protein expression, but to a lesser extent than NP alone. This behavior could be due to a possible competition between NP and the endogenous hormones for the same binding site. In this regard, the mixture-based approach is essential, since only combined exposure to multiple compounds allows for the emergence of antagonistic, additive, or synergistic interactions, effects that would remain undetectable when each substance is tested individually. Regarding ERβ, a progressive overexpression of the receptor was observed, especially following treatment with NP+T+E2. Dysregulation of the two estrogen receptor isoforms following treatment could lead to an alteration in cell susceptibility to endogenous hormones, particularly during the prostate’s developmental phase, and to the consequent genesis of organ disease.

The AR receptor, like the ERs, plays a pivotal role in the prostate, affecting both normal physiological function and the pathogenesis of prostate cancer. Once activated by androgens, it migrates to the nucleus, acting as a transcription factor for genes crucial to the development and maintenance of prostate tissue. In the presence of EDCs, AR signaling is dysregulated, contributing to prostate pathogenesis such as hyperplasia or prostate cancer. As with the ERs, we showed that AR protein expression had a transient alteration after short exposure to NP alone and in binary or tertiary mixtures with the endogenous hormones. After 2 h, NP alone did not affect AR expression. In contrast, NP+E2 markedly downregulated AR levels, suggesting that this mixture can alter the physiological receptor homeostasis. After 30 min and 4 h, all experimental classes induced a downregulation of AR compared with the control. AR downregulation induced by NP in mixture with endogenous hormones could lead to a lack of cellular responsiveness to one of the key hormones for prostate development and physiology.

As demonstrated in previous studies, NP interacted with the prostate receptors ERα, ERβ and AR, affecting their localization [23]. A crucial feature emerging from our data is the ability of the binary and ternary mixtures to retain ERα within the nucleus for prolonged periods. Rather than simply inducing receptor translocation, the mixtures promoted persistent nuclear localization. Whereas physiological E2 signaling is characterized by temporary, tightly timed receptor translocation, the mixtures shifted ERα toward a state of prolonged nuclear localization. The persistent ERα nuclear retention may result in its aberrant and prolonged activation, potentially triggering cellular responses at an inappropriate stage of the signaling cycle. None of the individual compounds, except E2, induced ERβ translocation. In contrast, all NP-containing mixtures promoted ERβ nuclear localization, with the most persistent effect observed after NP+E2 treatment. In the prostate, the two isoforms are involved in opposite processes: ERα principally mediates proliferative and pro-inflammatory responses, whereas ERβ promotes differentiation and apoptosis and exerts anti-inflammatory, tumor-suppressive effects, so the ERα/ERβ ratio plays a key role in prostate homeostasis [36]. Notably, under the NP+E2 treatment, a permanent nuclear localization of the two isoforms was observed. These findings raise the possibility that ERα and ERβ cooperate under NP+E2 exposure. Despite their opposite physiological functions, the overall response remained proliferative, suggesting that receptor cooperation predominates over functional antagonism. Further studies will be required to verify this hypothesis. Regarding AR, it showed a cyclic translocation after T treatment, locating the nucleus after 30 min and 2 h and returning to the cytoplasm after 4 h. NP did not fully mimic T: NP alone triggered AR translocation after 30 min but also forced the receptor into the nucleus after 4 h. NP+E2 induced AR nuclear translocation after 30 min and 2 h, closely resembling the effect of T. This finding is particularly intriguing because it suggests that an estrogenic mixture can activate a canonical androgen-dependent signaling pathway. Intriguingly, the NP+T mixture did not show AR translocation into the nucleus, suggesting a potential competitive interaction between NP and T during the canonical AR translocation process; this underlines the ability of NP to block AR in the cytoplasm, thereby rendering the cells unresponsive to androgenic stimulation. However, further investigation will be needed to confirm this hypothesis.

Finally, these substances also affected the cell migration rate: NP, both alone and in binary and tertiary mixtures with E2 and T, reduced cell migration, whereas in the presence of E2 or T alone, cell migration was higher than in the control, indicating a possible interference of NP with the processes involved in cytoskeletal remodeling during cell migration. We can hypothesize that, under physiological conditions, NP increases cell metabolic activity while blocking cell migration, making cells prone to hyperplasia and an inflammatory state; indeed, as previously shown, NP induced an increase in *IL-8* and *IL-1β* in PNT1A cells [23]. This finding highlights a dual role for NP: while cell migration was negatively influenced by NP under physiological conditions, under pathological conditions, it could enhance cell migration by increasing the expression of markers such as snail and vimentin; these effects are mediated through the estrogen receptor and can be inhibited by specific antagonists such as ICI 182,780 [36,37]. These observations are particularly relevant in the context of NP reproductive toxicity. NP has been shown to impair neonatal testicular development in mouse organ culture [13] and has been associated with male urogenital malformations such as hypospadias [14], indicating that the male reproductive tract is a sensitive target of this contaminant. However, most of these studies examined the male gonad and were based on exposure to NP alone. By focusing on the prostate, a hormone-dependent accessory gland central to male reproductive physiology and on its response to NP in combination with endogenous steroids, our work broadens the current understanding of NP reproductive toxicity beyond the testis and beyond single-compound exposure, addressing a district and an exposure scenario that had remained largely unexplored. To our knowledge, this is one of the first *in vitro* studies to examine an alkylphenol not in isolation, but against the physiological background of circulating steroid hormones. Earlier combined-exposure work on the prostate paired NP with a second xenestrogen, such as bisphenol A, in RWPE-1 cells [22], thus modeling the interaction between two contaminants. Yet NP never acts on a hormone-free background *in vivo:* it invariably meets the circulating estrogens and androgens that govern prostate homeostasis. Our data show that, under these conditions, NP interferes with the physiological action of these hormones, antagonizing the proliferative effect of E2 in the NP+E2 mixture, preventing the androgen-driven nuclear translocation of AR in the NP+T mixture, and counteracting the promigratory effect of both E2 and T. By capturing these interactions, our study provides both a mechanistic framework and an experimental paradigm for investigating EDCs within the hormonal milieu they are destined to perturb, offering a starting point for future *in vitro* and *in vivo* studies of contaminant–hormone interactions in reproductive tissues. This study has some limitations. First, a single non-tumorigenic prostate cell line (PNT1A) was used; although this is a well-established model for prostate endocrine disruption, additional prostate cell types and *in vivo* models will be needed to confirm the findings. Second, the mixtures were tested using a single NP concentration (10^−6^ M): although this dose corresponds to the concentration at which NP significantly affected cell viability, it is higher than the levels usually reported in environmental matrices, and the mixture results should therefore be regarded as a high-exposure (“worst-case”) scenario, useful to clarify the interaction between NP and steroid receptors rather than to model environmentally realistic co-exposure. Third, the experimental endpoints were assessed over different time points: receptor expression and localization were assessed at early time points (30 min, 2 and 4 h), whereas viability and migration were evaluated at 24 h. This reflects the different biology of the two types of analysis: receptor translocation and their expression levels are an early and transient event, whereas changes in viability and migration are cumulative effects that need longer exposure to appear. Fourth, cell viability was assessed by the MTT assay, which measures mitochondrial metabolic activity and therefore does not discriminate between an increase in cell number and an increase in metabolic activity per cell. Moreover, in the wound healing assay, a contribution of cell division to wound closure cannot be formally excluded. This is unlikely to explain our results for the mixtures: NP+T and NP+T+E2 increased the MTT signal but reduced wound closure, and a higher cell number would therefore have masked, rather than produced, the observed inhibition. In contrast, the increased closure induced by E2 and T alone should be interpreted with caution, since these treatments also increased cell viability. Future studies using lower NP concentrations, additional cell models, and matched time points, together with proliferation-specific assays such as cell cycle analysis or Ki-67 immunodetection, will be needed to confirm whether the proliferative effect suggested by the MTT data is retained by the mixtures, and will help clarify the environmental relevance of these interactions and the correlation between molecular and functional data.

## 5. Conclusions

This study confirms NP as an EDC, not only owing to its ability to act at low, non-monotonic concentrations, but also because it interacts with androgen and estrogen receptors, eliciting hormone-like cellular responses. Crucially, our data emphasize that NP retains its disrupting potential when combined with endogenous hormones: while NP alone increased cell viability, the NP+E2 mixture abolished this effect, revealing an antagonistic interaction; at the receptor level, the mixtures prolonged ERα nuclear retention and altered AR translocation in a compound-specific manner, and NP further counteracted the promigratory effect of E2 and T. This highlights a cocktail effect, particularly relevant because NP never acts alone *in vivo*, but always against a background of circulating endogenous hormones, potentially favoring the genesis of hormone-dependent pathologies, including prostate cancer. Our results provide a significant basis for elucidating cell-specific mechanisms, which will help refine experimental designs in future *in vivo* research.

## Figures and Tables

**Table 1:** Experimental classes used in this study (NP, 4-nonylphenol; E2, 17β-oestradiol; T, testosterone).

Experimental Classes	NP	E2	T
NP	10^−6^ M	-	-
E2	-	10^−6^ M	-
T	-	-	10^−8^ M
NP+E2	10^−6^ M	10^−6^ M	-
NP+T	10^−6^ M	-	10^−8^ M
T+E2	-	10^−6^ M	10^−8^ M
NP+T+E2	10^−6^ M	10^−6^ M	10^−8^ M

**Table 2:** Antibodies used for the Western blot assay.

**Primary Antibodies**	**Company, Cat. No.**	**Dilution**
Rabbit polyclonal ERα	Abcam, ab3575	1:500
Rabbit polyclonal ERβ	Sigma-Aldrich, SAB4500814	1:750
Rabbit polyclonal AR	Abcam, ab74272	1:1000
Rabbit polyclonal GAPDH	Elabscience, E-AB-40516	1:2000
**Secondary Antibodies**	**Company, Cat. No.**	**Dilution**
Goat anti-rabbit IgG (H&L), HRP conjugate	ImmunoReagent, GtxRb-003-DHRPX	1:2000

**Table 3:** Antibodies used for the immunofluorescence assay.

**Primary Antibodies**	**Company, Cat. No.**	**Dilution**
Rabbit polyclonal ERα	Abcam, ab3575	1:200
Rabbit polyclonal ERβ	Sigma-Aldrich, SAB4500814	1:300
Rabbit polyclonal AR	Abcam, ab74272	1:300
**Secondary Antibodies**	**Company, Cat. No.**	**Dilution**
Goat anti-rabbit IgG (H&L), DyLight 594 conjugate	ImmunoReagent, GtxRb-003-D594NHSX	1:300

## Data Availability

The data presented in this study are available on request from the corresponding author.

## Supplementary Materials

The following supporting information can be downloaded at https://www.mdpi.com/article/10.3390/toxics14090755/s1. Figure S1: Representative ERβ immunofluorescence images in PNT1A cells following single-compound treatments (NP, E2, T) at 30 min, 2 h and 4 h; Figure S2: Nuclear-to-cytoplasmic (N/C) fluorescence ratio of ERα, ERβ and AR in PNT1A cells after 30 min, 2 h and 4 h of treatment with the single compounds (NP, E2, T) and their mixtures (NP+E2, NP+T, T+E2, NP+T+E2) versus control. Additional supporting images are also provided: full-length Western blot membranes (including molecular weight markers and loading controls) and representative wound healing assay images for all experimental conditions.

## Abbreviations

The following abbreviations are used in this manuscript:

NP	4-Nonylphenol
E2	17β-Oestradiol
T	Testosterone
ER	Estrogen receptor
AR	Androgen receptor
EDC	Endocrine-disrupting chemical
SVHC	Substance of very high concern
MTT	3-(4,5-Dimethylthiazol-2-yl)-2,5-diphenyltetrazolium bromide
DAPI	4′,6-Diamidino-2-phenylindole
FBS	Fetal bovine serum
DMSO	Dimethyl sulfoxide
OD	Optical density
SEM	Standard error of the mean
